# Impact of the 2024 Resident Physician Work Stoppage on Acute Hemorrhagic Stroke Admissions: A Single Cerebrovascular-Specialty Hospital Study in South Korea

**DOI:** 10.3390/healthcare13172129

**Published:** 2025-08-27

**Authors:** Youngsoo Kim, Dougho Park, Haemin Kim, Dahyeon Koo, Sukkyoung Lee, Yejin Min, Daeyoung Hong, Mun-Chul Kim

**Affiliations:** 1 Department of Neurosurgery, Pohang Stroke and Spine Hospital, Pohang 37659, Republic of Korea; youngsooooo@gmail.com (Y.K.); soosungzzang42@gmail.com (H.K.); hongdy2000@gmail.com (D.H.); 2 Medical Research Institute, Pohang Stroke and Spine Hospital, Pohang 37659, Republic of Korea; parkdougho@gmail.com (D.P.); dahyeonkoo@gmail.com (D.K.); kyoung821130@gmail.com (S.L.); alsdpwls777@gmail.com (Y.M.); 3 Medical Science and Engineering, Graduate School of Convergence Science and Technology, Pohang University of Science and Technology, Pohang 37666, Republic of Korea

**Keywords:** health care delivery, health services accessibility, hemorrhagic stroke, special hospitals, time-to-treatment

## Abstract

**Background**: In February 2024, a nationwide resignation of resident physicians and fellows in South Korea caused a sudden disruption in the healthcare service delivery system. This study aimed to investigate how the crisis affected hospital admission patterns, treatment timelines, and early outcomes in patients with acute hemorrhagic stroke. **Methods**: We retrospectively analyzed data from prospective cohorts of patients diagnosed with intracerebral hemorrhage or subarachnoid hemorrhage admitted to a single cerebrovascular-specialty hospital between March 2023 and February 2025. Patients were categorized into two groups: those admitted before (Before crisis group, *n* = 130) and after (After crisis group, *n* = 214) the crisis. Clinical characteristics, regional distribution, time delays, and 3-month modified Rankin Scale (mRS) outcomes were compared. **Results**: Following the crisis, a significant increase was observed in admissions from outside the hospital’s primary coverage area (*p* < 0.001). Onset-to-arrival (138.0 vs. 92.0 min, *p* = 0.040) and onset-to-operation times (200.0 vs. 166.0 min, *p* = 0.046) were significantly delayed, particularly in patients who underwent surgical treatment. However, arrival-to-operation time remained stable (*p* = 0.694), and initial neurological severity was comparable. Functional outcomes at 3 months did not differ significantly (mRS 0–2: 53.8% vs. 50.5%, *p* = 0.157), indicating preserved in-hospital care quality, despite external disruption. **Conclusions**: The medical crisis disrupted the stroke care delivery system and delayed prehospital care in South Korea. Nevertheless, the cerebrovascular-specialty hospital maintained timely intervention and preserved outcomes. These findings support the strategic importance of decentralized specialty hospitals in ensuring the resilience of the healthcare service delivery system during a national healthcare crisis.

## 1. Introduction

Acute hemorrhagic stroke, including intracerebral hemorrhage (ICH) and subarachnoid hemorrhage (SAH), is among the most severe forms of stroke, often resulting in high mortality rates and long-term disability [1,2]. Timely diagnosis and intervention are crucial, as delays in initial management can lead to irreversible neurological damage and, ultimately, increased healthcare burden [3]. Therefore, timely and adequate intervention remains a critical component of organized stroke care delivery [4,5].

In South Korea, the national healthcare delivery system for acute critical care remains heavily reliant on large tertiary hospitals [6]. However, persistent disparities in healthcare resource distribution and shortages in essential critical care personnel have created systemic vulnerabilities [7,8]. Moreover, the nationwide medical crisis that began in early 2024 worsened these systemic imbalances. The South Korean healthcare system faced an unprecedented shock after the government announced a plan to increase medical school enrollment by 2000 students annually—citing projected physician shortages—and over 90% of surgical trainees and other residents resigned from more than 100 hospitals [9,10,11]. The Korean Medical Association immediately criticized the policy as misaligned with structural issues of resource distribution and training quality [12]. However, the main issue was that this professional resistance led to reduced service capacity across major tertiary hospitals, affecting emergency, surgical, and critical care departments [13]. Consequently, this unprecedented crisis raised serious concerns about the stability and resilience of the country’s infrastructure for managing acute and critical conditions, including acute hemorrhagic stroke [14,15].

To address these longstanding challenges in the distribution of critical care resources, the Ministry of Health and Welfare in South Korea implemented the Specialty Hospital Designation Program, in 2011 [16,17]. This initiative aimed to mitigate the overconcentration of patients in tertiary general hospitals and promote equitable regional access to healthcare [18]. Hospitals are designated as specialty centers by the Korean Health Insurance Review and Assessment Service, based on strict criteria regarding infrastructure, patient composition, and staffing [19]. Within this framework, cerebrovascular-specialty hospitals have emerged as an essential component of South Korea’s stroke care system. These hospitals are required to perform complex procedures such as open cranial surgeries, including intracranial and intraventricular hematoma evacuation, decompressive craniectomy, and endovascular interventions, including mechanical thrombectomy, intravascular stenting, and aneurysm coiling, while maintaining the capacity for comprehensive and timely stroke management, which is substantially demanding [20]. Their designation serves not only to support high-volume, high-acuity stroke care, but also to function as decentralized nodes in the national emergency care network.

Prior studies have examined the impact of pandemic crises, such as the coronavirus disease (COVID-19) pandemic, on stroke systems of care [21]; however, the effect of non-infectious healthcare crises, particularly workforce-related disruptions, on acute stroke delivery remains scarce. To our knowledge, no study has evaluated the regional redistribution or time-related features in stroke care associated with a nationwide physician labor action. In this context, we aimed to examine the impact of the 2024 national medical crisis—specifically the mass resignation of resident physicians—on the delivery of acute hemorrhagic stroke care in South Korea. This study sought to (1) assess changes in patient admission patterns, including regional distribution and referral sources; (2) evaluate delays in critical time-to-treatment metrics; and (3) compare short-term functional outcomes before and after the onset of the crisis. We hypothesized that the abrupt depletion of frontline surgeons and physicians at tertiary centers resulted in diminished emergency department capacity, prolonged triage times, and the eventual diversion of patients to more distant facilities, including specialty hospitals. This conceptual pathway underlies the redistribution of patient inflow and the observed treatment delays, and serves as the basis for our system-level analysis.

## 2. Materials and Methods

### 2.1. Participants and Study Design

This study retrospectively analyzed the data of prospective stroke cohorts—the Korean Stroke Registry (PSSH0475-2021-08-HR-016) and the Korean Hemorrhagic Stroke Registry (PSSH0475-202304-HR-007)—collected at a single, regional cerebrovascular-specialty hospital in South Korea. The initial sample included patients who were admitted with a primary diagnosis of acute ICH or SAH between March 2023 and February 2025. The diagnoses of ICH and SAH were identified based on the International Classification of Diseases, 10th Revision (ICD-10), codes I61 and I60, respectively.

**Figure 1:**
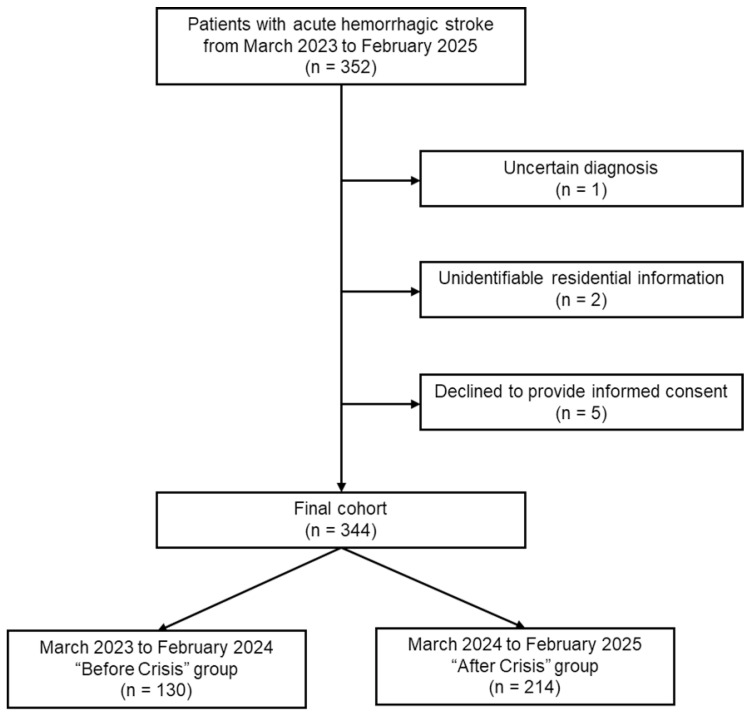
Flow chart of this study.

The exclusion criteria were as follows: (1) the diagnosis of ICH or SAH was uncertain (*n* = 1); (2) the patient’s residential information was missing or unidentifiable (*n* = 2); or (3) the patient declined to provide informed consent (*n* = 5). Ultimately, 344 patients were included in the final cohort. Patients were stratified into two groups, according to the time of admission relative to the national medical crisis: those admitted between March 2023 and February 2024 were categorized as the “Before Crisis” (BC) group (*n* = 130) and those admitted between March 2024 and February 2025 as the “After Crisis” (AC) group (*n* = 214) (Figure 1).

This study protocol was reviewed and approved by the Institutional Review Board (PSSH0475-202304-HR-007-04), and informed consent was obtained from all participants. To ensure confidentiality, the Institutional Review Board required and approved a de-identification process in which patients’ residential information was coded separately from clinical data and provided to researchers as an independent dataset.

### 2.2. Variables

Baseline demographic and clinical characteristics, including age, sex, body mass index, region of residence, visit type, and smoking status, were collected at the time of admission. Comorbidities such as hypertension, diabetes mellitus, dyslipidemia, coronary artery disease, and prior cerebrovascular events were recorded according to the medical record or self-report.

Neurological severity at admission was assessed using the Glasgow Coma Scale (GCS) and the National Institutes of Health Stroke Scale (NIHSS). Time-related metrics were calculated as follows: times from symptom onset to hospital arrival (onset-to-arrival time), from symptom onset to surgical intervention (onset-to-operation time), and from hospital arrival to initiation of surgery (arrival-to-operation time). Surgical treatment was defined as any of the following: intracranial and intraventricular hematoma evacuation, decompressive craniectomy, or endovascular interventions.

Functional outcomes were measured using the modified Rankin Scale (mRS) three months after stroke onset. All outcome assessments were based on clinician reviews and follow-up records.

### 2.3. Statistical Analysis

Normality of continuous variables was assessed using the Kolmogorov–Smirnov test. Variables with normal distributions are presented as mean ± standard deviation, while those without are expressed as median with interquartile range. Group comparisons were performed using the independent *t*-test or the Wilcoxon rank-sum test, as appropriate. Categorical variables are expressed as frequency (percentage), and differences between groups were evaluated using the chi-squared test. Statistical significance was set at *p* < 0.05. All analyses were conducted using complete case analysis. For sensitivity analysis, subgroup stratification was performed according to primary diagnosis (ICH or SAH), and the decision was made whether or not to apply surgical treatments. We additionally performed a 1:1 propensity score matching as a sensitivity analysis, using nearest neighbor matching without replacement. Matching variables included age, sex, and primary diagnosis. All matched analyses were conducted using the MatchIt package. All statistical analyses were performed using R software version 4.5.0 (R Core Team, Vienna, Austria).

## 3. Results

### 3.1. Baseline Characteristics

Table 1 presents the baseline characteristics of patients. No significant difference was observed between groups regarding age, sex, body mass index, or comorbidities. Neurological severity at admission, assessed by the GCS and NIHSS, did not differ significantly between groups. Approximately 59% of patients in each group underwent surgical intervention. Hospital stay durations were similar. Initial vital signs and laboratory findings of the patients did not differ between groups (Table S1).

**Figure 2:**
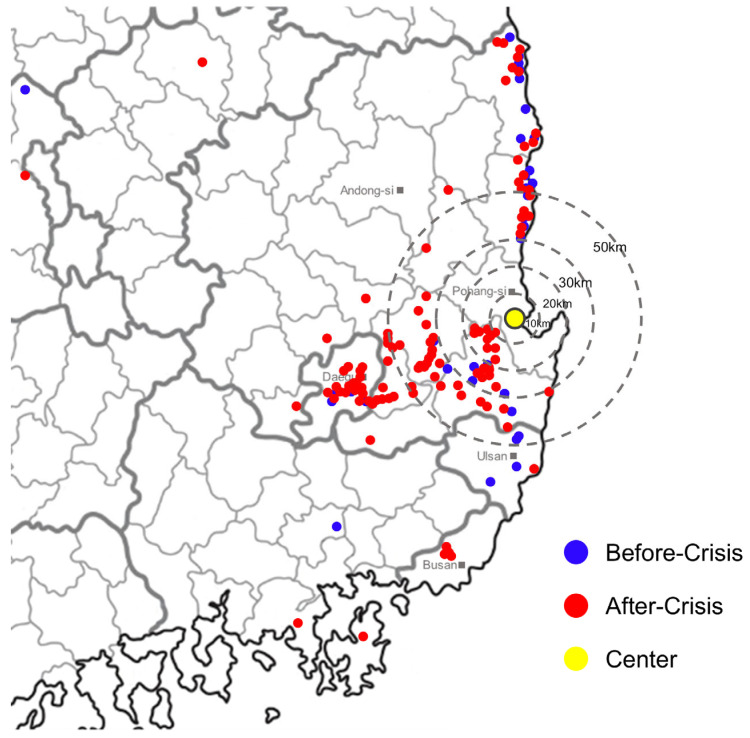
Regional distribution of patients from the hospital (yellow spot). Following the medical crisis, a significant number of patients began arriving from outside the hospital’s regional coverage area (red spots), particularly from the metropolitan area of Daegu and Gyeongsangbuk Province. Concentric rings at 10, 20, 30, and 50 km serve as visual references only.

**Figure 3:**
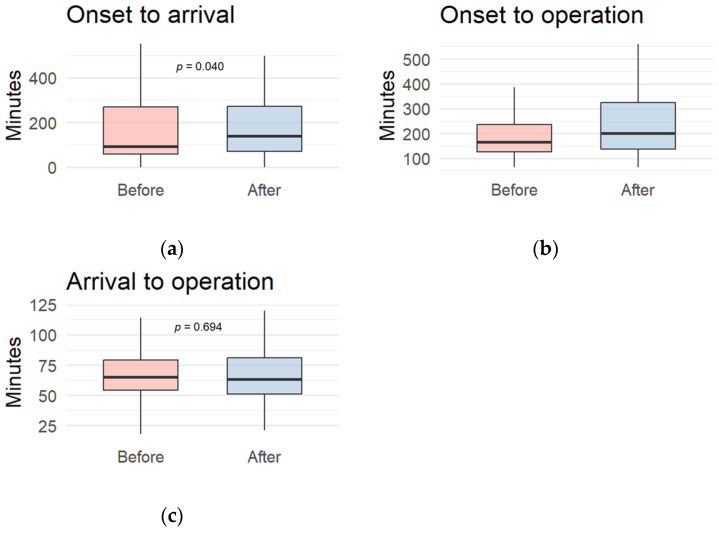
Comparisons of time-related parameters. Significant delays of (**a**) onset-to-arrival and (**b**) onset-to-operation times were observed after the medical crisis. However, no difference was observed in (**c**) arrival-to-operation time. *p*-values were calculated using Wilcoxon rank-sum test.

The proportion of patients diagnosed with ICH was higher in the BC group (77.7%) than in the AC group (67.3%), whereas the proportion of patients diagnosed with SAH increased after the crisis (22.3% vs. 32.7%, *p* = 0.052). A significant shift was observed in the regional distribution: patients admitted from outside the hospital’s primary coverage area increased after the crisis (*p* < 0.001), particularly from the metropolitan Daegu and north-west Gyeongsangbuk provinces, which are relatively far from Pohang city (Figure 2). This shift in regional distribution remained statistically significant, even after propensity score matching by age, sex, and primary diagnosis (*p* < 0.001) (Table S2). Referral rates also increased, although the difference was not statistically significant (23.8% vs. 33.2%, *p* = 0.126). Significant delays were observed in two key time intervals: onset-to-arrival time increased from 92.0 (57.0–272.0) to 138.0 (69.0–273.0) min (95% confidence interval [CI]: −58.5; 77.9, *p* = 0.040), and onset-to-operation time increased from 166.0 (125.0–235.0) to 200.0 (135.0–325.0) min (95% [CI]: −45.4; 101.6, *p* = 0.046). Meanwhile, arrival-to-operation time remained unchanged (65.0 vs. 63.0 min, 95% CI: −14.0; 7.3, *p* = 0.694) (Figure 3).

**Figure 4:**
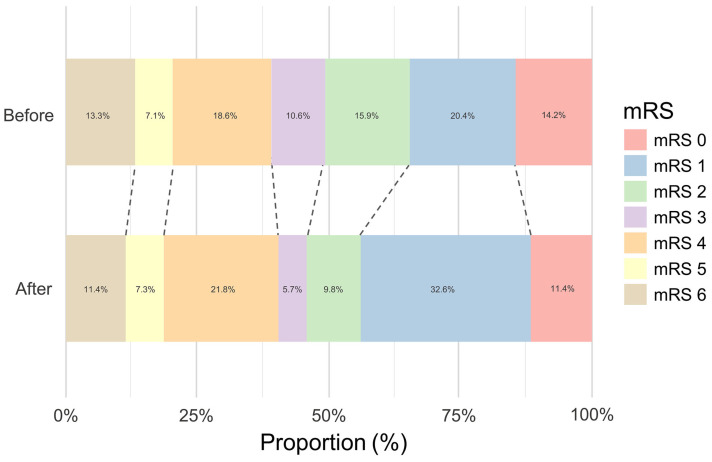
Functional outcome after 3 months. No significant difference was observed between groups. Abbreviation: mRS, modified Rankin scale.

At the 3-month follow-up, functional outcomes, as measured by the mRS, were comparable across groups (*p* = 0.157). The proportion of patients achieving mRS 0–2 was 50.5% in the BC group and 53.8% in the AC group (Figure 4).

### 3.2. Subgroup Analysis According to Primary Diagnosis

Among patients with ICH, a significant delay was observed in the onset-to-operation interval in the AC group compared to the BC group (185.0 vs. 145.0 min, *p* = 0.017), whereas arrival-to-operation times remained similar. The regional redistribution pattern was more prominent in the AC group, with a higher proportion of patients originating outside the primary hospital coverage area (*p* < 0.001). Functional outcomes at 3 months did not differ significantly between the groups (*p* = 0.597) (Table 2).

In contrast, patients with SAH showed no significant differences in any time interval or baseline characteristics between groups. However, regional shifts were again observed (*p* = 0.018), with more patients referred from distant areas after the crisis. Functional outcomes remained comparable (Table S3).

### 3.3. Subgroup Analysis According to Surgical Treatment

Among patients who underwent surgical treatment, both onset-to-arrival (135.5 vs. 85.0 min, *p* = 0.013) and onset-to-operation times (200.0 vs. 166.0 min, *p* = 0.046) were significantly prolonged after the crisis, despite stable arrival-to-operation intervals. No significant differences were found in initial severity or functional outcomes (Table 3).

In non-operated patients, baseline characteristics were generally similar. Geographic redistribution and referral patterns shifted significantly after the crisis (*p* = 0.028 and *p* = 0.016, respectively). However, no significant time delays or differences were noted in mRS outcomes (Table S4).

## 4. Discussion

In this study, we analyzed the impact of a nationwide medical crisis on the admission patterns and treatment timelines of patients with acute hemorrhagic stroke at a cerebrovascular-specialty hospital in South Korea. We observed a significant increase in prehospital delays following the crisis, particularly in more severe cases that required surgical intervention. In addition, there was a notable shift in the regional distribution of admissions, with more patients coming from outside the hospital’s usual coverage area. These findings suggest that the national healthcare service delivery system has experienced a significant dysfunction in response to the medical crisis, including the acute stroke care services.

Studies related to this unusual medical crisis have been reported in South Korea [14,22]. Cho et al. [23] reported that a medical crisis significantly impacted research activities, leading to a significant decrease in medical research after the crisis. Lee et al. [24] reported that despite the medical crisis in South Korea, patient outcomes for gastric cancer surgery were comparable; however, the interpretation of the results should be approached with caution, owing to the reduced number of surgeries and the limited data collection period between March and May 2024. To our knowledge, our study is the first to evaluate the pattern and outcomes of acute hemorrhagic stroke admissions during a relatively prolonged period after the medical crisis and to elucidate a system-level healthcare service dysfunction triggered by the unusual situation in South Korea, representing the great strength of our results.

Furthermore, our findings suggest a dysfunction in the stroke care delivery system during the medical crisis. Notably, there was a marked increase in the admissions of patients with acute hemorrhagic stroke from outside the hospital’s primary coverage area during the post-crisis period. This redistribution pattern was consistently observed across subgroups, indicating a systemic disruption, rather than isolated variation. Our sensitivity analyses further supported the robustness of our findings. In addition, consistent and statistically significant delays were observed in both onset-to-arrival and onset-to-operation times among patients who underwent surgical intervention, typically representing more severe cases. These delays suggest that even critical cases were affected by reduced accessibility or inefficiencies in the emergency care routine.

Despite these challenges, functional outcomes at 3 months were preserved, and initial neurological severity did not differ significantly between the groups. Arrival-to-operation time remained stable, suggesting that appropriate and timely care was delivered once patients arrived at the hospital. This emphasizes the resilience and effectiveness of specialized hospitals in maintaining care quality during the post-crisis period. Currently, there are only four officially designated cerebrovascular-specialty hospitals in South Korea [21]; these institutions offer an alternative to the tertiary hospital-centered model by providing regionally accessible, high-level stroke care [25]. Nevertheless, the current national stroke care policy remains focused on large, tertiary hospitals [26,27], which may lack the flexibility to effectively respond to regional system failures. Our findings highlight the need to re-evaluate and strengthen the role of cerebrovascular-specialty hospitals. These centers were less susceptible to external disruptions and maintained clinical performance, despite increased patient burden. Consequently, strategic investment in, and expansion of, such specialized hospitals may enhance the resilience of stroke care services under future crises.

Furthermore, their structural independence and specialized focus may render them less vulnerable to systemic shocks, such as national healthcare crises or pandemics. Evidence suggests that such specialty hospitals can maintain quality care and treatment timelines, even during the COVID-19 pandemic [28]. During that time, many countries and centers reported reduced cases of acute stroke, delayed care processes, and lower rates of acute vascular treatment [29,30]. For example, a study in China reported an approximately 40.0% reduction in stroke admissions and a 25% reduction in thrombolysis and thrombectomy cases during the pandemic [11]. In addition, a study from German centers also reported decreased hospital admissions owing to cerebrovascular events, resulting from social distancing and health care restrictions [31]. Meanwhile, a South Korean cerebrovascular specialty hospital report based on the Korean Stroke Registry reported that the number of acute ischemic stroke cases, the admission process times, and the rates and rapidness of acute vascular treatment did not deteriorate during the COVID-19 pandemic [25]. Our findings further validate the critical role of specialized hospitals in ensuring timely assessment and treatment for acute conditions during a national medical crisis and the healthcare service disparities regarding essential critical care, taking into account the characteristics of the South Korean healthcare service [32].

Without structural changes in the emergency delivery system, future strikes, staffing disruptions, or pandemic crises may jeopardize timely care for critical conditions such as stroke. Our findings suggest that strengthening decentralized care systems—through strategic support and expansion of cerebrovascular-specialty hospitals—could mitigate the effects of such crises. In parallel, policies that enable surge staffing, regional coordination, and transparent workforce planning may enhance systemic resilience. Additionally, prehospital triage protocols should be dynamically adjusted to reflect hospital functionality and real-time capacity, particularly for time-sensitive conditions such as hemorrhagic stroke. Collaboration between emergency medical services and regional specialty hospitals should be institutionalized, including the development of shared dispatch systems and bypass policies during times of system stress [33,34]. Anticipating and preparing for recurring disruptions is essential for protecting vulnerable patient populations and maintaining equitable access to acute care.

This study has some limitations. First, data were obtained from a single institution, which may limit the generalizability of the findings. Second, the retrospective and descriptive design restricts causal inferences. Nevertheless, this study provides insights into the real-world effects of a nationwide medical crisis on a highly time-sensitive disease, and highlights the importance of specialized care models. Third, we did not consider seasonal variation or the time distribution of the day in relation to acute hemorrhagic stroke occurrence in our analysis, as was noted in previous studies [35]. Fourth, the possibility of survivor bias must be considered. Patients who survived longer transport times may represent a selective subset with better baseline physiology, potentially exaggerating our outcomes. Fifth, we did not utilize emergency medical service-level data, such as dispatch intervals, traffic conditions, or distance traveled. These unmeasured variables may have influenced our results. Finally, while our findings support the expansion of cerebrovascular-specialty hospitals, we acknowledge that such policy shifts must be balanced against potential trade-offs. These include increased capital investment, the risk of duplicating high-acuity services, and the possibility of workforce redistribution from tertiary centers. Future policy planning should consider these aspects, to ensure resource optimization within the broader national healthcare framework.

## 5. Conclusions

The 2024 national medical crisis in South Korea was associated with increased prehospital delays and changes in the regional distribution of patients with acute hemorrhagic stroke. Despite these challenges, a cerebrovascular-specialty hospital maintained timely in-hospital treatment and preserved functional outcomes. These findings underscore the resilience and utility of specialized stroke care centers during healthcare system stress, and suggest that nurturing and expanding cerebrovascular-specialty hospitals may strengthen the flexibility and responsiveness of national stroke care systems. Furthermore, findings of this study support the broader vision of developing an integrated and inclusive acute stroke care system—one in which tertiary centers, specialty hospitals, and regional emergency medical systems collaborate seamlessly to provide timely, high-quality care to all patients, even during a national healthcare crisis.

## Figures and Tables

**Table 1:** Demographic characteristics of patients.

Variables	BC Group (*n* = 130)	AC Group (*n* = 214)	*p*-Value ^e^
Age, years	63.0 (51.0–75.0)	65.0 (55.0–76.0)	0.348
Male, n (%)	65 (50.0)	101 (47.2)	0.694
Body mass index, kg/m^2^	23.6 (20.3–25.7)	23.7 (21.3–25.8)	0.570
Primary diagnosis, n (%)			0.052
Intracranial hemorrhage	101 (77.7)	144 (67.3)	
Subarachnoid hemorrhage	29 (22.3)	70 (32.7)	
Regional categories, n (%)			<0.001
Within Pohang city	63 (48.5)	75 (35.0)	
Within the region ^a^	54 (41.5)	66 (30.8)	
Within Daegu/Gyeongsangbuk province ^b^	8 (6.2)	63 (29.4)	
Outside Daegu/Gyeongsangbuk province	5 (3.8)	10 (4.7)	
Visit types, n (%)			0.126
First visit	89 (68.5)	133 (62.1)	
Transferred from another hospital ^c^	10 (7.7)	10 (4.7)	
Referred from another hospital ^d^	31 (23.8)	71 (33.2)	
Initial GCS score	14.0 (9.0–15.0)	15.0 (9.0–15.0)	0.388
NIHSS	8.0 (1.0–19.0)	6.0 (0.0–16.0)	0.129
Operation, n (%)	77 (59.2)	126 (58.9)	>0.999
Onset-to-arrival, minutes	92.0 (57.0–272.0)	138.0 (69.0–273.0)	0.040
Onset-to-operation, minutes	166.0 (125.0–235.0)	200.0 (135.0–325.0)	0.046
Arrival at initial imaging, minutes	16.0 (11.0–28.0)	15.0 (11.0–25.0)	0.267
Arrival-to-operation, minutes	65.0 (54.0–79.0)	63.0 (51.0–81.0)	0.694
Hospital stay, days	23.0 (12.0–35.0)	21.0 (12.0–31.0)	0.425
Current smoker, n (%)	33 (25.4)	46 (21.6)	0.499
Comorbidities, n (%)			
Hypertension	58 (44.6)	100 (46.7)	0.787
Diabetes	25 (19.2)	36 (16.8)	0.673
Dyslipidemia	21 (16.2)	45 (21.0)	0.331
Coronary artery diseases	11 (8.5)	14 (6.5)	0.652
Cerebrovascular accidents	24 (18.5)	36 (16.8)	0.809
Modified Rankin scale at 3 months, n (%)			0.157
0	16 (14.2)	22 (11.4)	
1	23 (20.4)	63 (32.6)	
2	18 (15.9)	19 (9.8)	
3	12 (10.6)	11 (5.7)	
4	21 (18.6)	42 (21.8)	
5	8 (7.1)	14 (7.3)	
6	15 (13.3)	22 (11.4)	

^a^ Gyeongju, Yeongdeok, and Uljin. ^b^ Excluding the Gyeongju, Yeongdeok, and Uljin areas. ^c^ ≥6 h stay. ^d^ <6 h stay. ^e^ *p*-values calculated using Wilcoxon rank-sum test for continuous variables and chi-squared test for categorical variables. Abbreviations: AC, after crisis; BC, before crisis; GCS, Glasgow Coma Scale; NIHSS, National Institutes of Health Stroke Scale.

**Table 2:** Characteristics of patients with intracranial hemorrhage.

Variables	BC Group (*n* = 101)	AC Group (*n* = 144)	*p*-Value ^e^
Age, years	63.0 (51.0–76.0)	68.0 (58.0–76.0)	0.174
Male, n (%)	57 (56.4)	81 (56.2)	>0.999
Body mass index, kg/m^2^	23.7 (20.9–26.0)	23.9 (21.3–26.2)	0.432
Regional categories, n (%)			<0.001
Within Pohang city	51 (50.5)	51 (35.4)	
Within the region ^a^	39 (38.6)	46 (31.9)	
Within Daegu/Gyeongsangbuk province ^b^	6 (5.9)	40 (27.8)	
Outside Daegu/Gyeongsangbuk province	5 (5.0)	7 (4.9)	
Visit types, n (%)			0.317
First visit	73 (72.3)	99 (68.8)	
Transferred from another hospital ^c^	9 (8.9)	8 (5.6)	
Referred from another hospital ^d^	19 (18.8)	37 (25.7)	
Initial GCS score	14.0 (9.0–15.0)	14.0 (9.0–15.0)	0.923
NIHSS	10.0 (3.0–19.0)	10.0 (2.5–19.0)	0.739
Operation, n (%)	49 (48.5)	65 (45.1)	0.696
Onset-to-arrival, minutes	92.0 (56.0–249.0)	136.0 (68.0–266.5)	0.053
Onset-to-operation, minutes	145.0 (115.0–200.0)	185.0 (140.0–300.0)	0.017
Arrival at initial imaging, minutes	17.0 (11.0–29.0)	15.0 (11.0–26.0)	0.451
Arrival-to-operation, minutes	60.0 (54.0–79.0)	68.0 (60.0–87.0)	0.108
Hospital stay, days	22.0 (11.5–34.5)	19.0 (11.0–29.5)	0.384
Current smoker, n (%)	26 (25.7)	27 (18.9)	0.262
Comorbidities, n (%)			
Hypertension	46 (45.5)	74 (51.4)	0.441
Diabetes	21 (20.8)	28 (19.4)	0.922
Dyslipidemia	15 (14.9)	31 (21.5)	0.250
Coronary artery diseases	9 (8.9)	12 (8.3)	>0.999
Cerebrovascular accidents	18 (17.8)	32 (22.2)	0.496
Modified Rankin scale at 3 months, n (%)			0.597
0	6 (7.0)	5 (4.0)	
1	16 (18.6)	31 (25.0)	
2	16 (18.6)	15 (12.1)	
3	9 (10.5)	10 (8.1)	
4	19 (22.1)	34 (27.4)	
5	7 (8.1)	13 (10.5)	
6	13 (15.1)	16 (12.9)	

^a^ Gyeongju, Yeongdeok, and Uljin. ^b^ Excluding the Gyeongju, Yeongdeok, and Uljin areas. ^c^ ≥6 h stay. ^d^ <6 h stay. ^e^ *p*-values were calculated using Wilcoxon rank-sum test for continuous variables and chi-squared test for categorical variables. Abbreviations: AC, after crisis; BC, before crisis; GCS, Glasgow Coma Scale; NIHSS, National Institutes of Health Stroke Scale.

**Table 3:** Characteristics of patients with operations.

Variables	BC Group (*n* = 77)	AC Group (*n* = 126)	*p*-Value ^e^
Age, years	61.0 (51.0–72.0)	64.0 (54.0–76.0)	0.103
Male, n (%)	36 (46.8)	52 (41.3)	0.536
Body mass index, kg/m^2^	24.2 (22.0–26.3)	23.4 (20.8–25.8)	0.281
Primary diagnosis, n (%)			0.125
Intracranial hemorrhage	49 (63.6)	65 (51.6)	
Subarachnoid hemorrhage	28 (36.4)	61 (48.4)	
Regional categories, n (%)			<0.001
Within Pohang city	36 (46.8)	41 (32.5)	
Within the region ^a^	32 (41.6)	37 (29.4)	
Within Daegu/Gyeongsangbuk province ^b^	5 (6.5)	41 (32.5)	
Outside Daegu/Gyeongsangbuk province	4 (5.2)	7 (5.6)	
Visit types, n (%)			0.067
First visit	55 (71.4)	70 (55.6)	
Transferred from another hospital ^c^	2 (2.6)	8 (6.3)	
Referred from another hospital ^d^	20 (26.0)	48 (38.1)	
Initial GCS score	12.0 (7.0–15.0)	13.0 (8.0–15.0)	0.241
NIHSS	14.0 (5.0–25.0)	10.0 (0.0–20.0)	0.050
Onset-to-arrival, minutes	85.0 (55.0–142.0)	135.5 (63.0–257.0)	0.013
Onset-to-operation, minutes	166.0 (125.0–235.0)	200.0 (135.0–325.0)	0.046
Arrival at initial imaging, minutes	13.0 (10.0–21.0)	13.0 (9.0–18.0)	0.280
Arrival-to-operation, minutes	65.0 (54.0–79.0)	63.0 (51.0–81.0)	0.694
Hospital stay, days	24.0 (16.0–40.0)	27.0 (18.0–37.0)	0.865
Current smoker, n (%)	18 (23.4)	24 (19.0)	0.575
Comorbidities, n (%)			
Hypertension	33 (42.9)	57 (45.2)	0.853
Diabetes	16 (20.8)	15 (11.9)	0.132
Dyslipidemia	15 (19.5)	25 (19.8)	>0.999
Coronary artery diseases	7 (9.1)	9 (7.1)	0.817
Cerebrovascular accidents	18 (23.4)	22 (17.5)	0.397
Modified Rankin scale at 3 months, n (%)			0.646
0	11 (17.5)	16 (13.6)	
1	11 (17.5)	28 (23.7)	
2	8 (12.7)	11 (9.3)	
3	5 (7.9)	6 (5.1)	
4	13 (20.6)	32 (27.1)	
5	4 (6.3)	11 (9.3)	
6	11 (17.5)	14 (11.9)	

^a^ Gyeongju, Yeongdeok, and Uljin. ^b^ Excluding the Gyeongju, Yeongdeok, and Uljin areas. ^c^ ≥6 h stay. ^d^ <6 h stay. ^e^ *p*-values were calculated using Wilcoxon rank-sum test for continuous variables and chi-squared test for categorical variables. Abbreviations: AC, after crisis; BC, before crisis; GCS, Glasgow Coma Scale; NIHSS, National Institutes of Health Stroke Scale.

## Data Availability

The raw data supporting the conclusions of this article will be made available by the authors on request.

## Supplementary Materials

The following supporting information can be downloaded at: https://www.mdpi.com/article/10.3390/healthcare13172129/s1, Table S1: Initial vital signs and laboratory findings of the patients; Table S2: Regional distribution and time-related parameters after propensity score matching; Table S3: Characteristics of patients with subarachnoid hemorrhage; Table S4: Characteristics of patients without any operative care.

## Abbreviations

The following abbreviations are used in this manuscript:

ICH	Intracerebral hemorrhage
SAH	Subarachnoid hemorrhage
COVID-19	coronavirus disease
ICD	International Classification of Diseases
BC	Before crisis
AC	After crisis
GCS	Glasgow coma scale
NIHSS	National Institutes of Health Stroke Scale
mRS	modified Rankin scale
95% CI	95% confidence interval

## References

[1] Magid-Bernstein J., Girard R., Polster S., Srinath A., Romanos S., Awad I.A., Sansing L.H. (2022). Cerebral hemorrhage: Pathophysiology, treatment, and future directions. Circ. Res..

[2] Wan A., Jaja B.N., Schweizer T.A., Macdonald R.L. (2016). Clinical characteristics and outcome of aneurysmal subarachnoid hemorrhage with intracerebral hematoma. J. Neurosurg..

[3] Cheon S., Lee H., Won J., Jang B.H., Wang J.D. (2020). Lifetime risks and health impacts of hemorrhagic and ischemic stroke in South Korea. Sci. Rep..

[4] Almubayyidh M., Alghamdi I., Parry-Jones A.R., Jenkins D. (2024). Prehospital identification of intracerebral haemorrhage: A scoping review of early clinical features and portable devices. BMJ Open.

[5] Agrawal D., Dhillon P., Siow I., Lee K.S., Spooner O., Yeo L., Bhogal P. (2025). Prehospital technologies for early stroke detection—A review. Interv. Neuroradiol..

[6] Lee J.Y., Jo M.W., Yoo W.S., Kim H.J., Eun S.J. (2014). Evidence of a broken healthcare delivery system in korea: Unnecessary hospital outpatient utilization among patients with a single chronic disease without complications. J. Korean Med. Sci..

[7] Eun S.J. (2022). Trends and disparities in avoidable, treatable, and preventable mortalities in South Korea, 2001-2020: Comparison of capital and non-capital areas. Epidemiol. Health.

[8] Kim J.Y., Kang K., Kang J., Koo J., Kim D.H., Kim B.J., Kim W.J., Kim E.G., Kim J.G., Kim J.M. (2019). Executive summary of stroke statistics in Korea 2018: A report from the epidemiology research council of the Korean stroke society. J. Stroke.

[9] Park J., Shin C.H., Lee J.Y. (2024). Why did all the residents resign? Key takeaways from the junior physicians’ mass walkout in South Korea. J. Grad. Med. Educ..

[10] The Lancet Regional Health-Western Pacific (2024). Junior doctor strikes in South Korea: More doctors are needed?. Lancet Reg. Health West Pac..

[11] Zhao J., Li H., Kung D., Fisher M., Shen Y., Liu R. (2020). Impact of the COVID-19 epidemic on stroke care and potential solutions. Stroke.

[12] Lee B.K. (2024). The dispute over increasing medical student numbers in South Korea. J. Korean Assoc. Oral. Maxillofac. Surg..

[13] Kim J.H., Lee S., Park K., Lee K.Y., Jang J.Y. (2025). Impact of resident shortage on trauma care during the 2024 medical conflict: A single regional emergency medical center experience and recommendations. J. Acute Care Surg..

[14] Yoon J.H., Kwon I.H., Park H.W. (2024). The South Korean health-care system in crisis. Lancet.

[15] Ministry of Health and Welfare, Government of Korea Emergency Briefing on the Physician Workforce Expansion Plan. https://www.mohw.go.kr/board.es?mid=a10503000000&bid=0027&list_no=1480186&act=view.

[16] Kim S.J., Lee S.G., Kim T.H., Park E.C. (2015). Healthcare spending and performance of specialty hospitals: Nationwide evidence from colorectal-anal specialty hospitals in South Korea. Yonsei Med. J..

[17] The Korean Ministry of Health and Welfare Designation of Specialty Hospital. https://www.mohw.go.kr/menu.es?mid=a10702010100.

[18] Lee K.S., Chun K.H., Lee J.S. (2008). Reforming the hospital service structure to improve efficiency: Urban hospital specialization. Health Policy.

[19] Kim S.J., Yoo J.W., Lee S.G., Kim T.H., Han K.T., Park E.C. (2014). Governmental designation of spine specialty hospitals, their characteristics, performance and designation effects: A longitudinal study in Korea. BMJ Open.

[20] Bang J.S. (2022). Vulnerable shadows in splendid Korean big hospitals. J. Korean Med. Sci..

[21] Al Hashmi A., von Bandemer S., Shuaib A., Mansour O.Y., Wassy M., Ozdemir A.O., Farhoudi M., Al Jehani H., Khan A., John S. (2022). Lessons learned in stroke care during COVID-19 pandemic and preparing for future pandemics in the MENA+ region: A consensus statement from the MENA+–SINO. J. Neurol. Sci..

[22] de Beer A., Werner A.S., Kim S., Jenne F.A. (2024). Professional resistance: Why Korean medical students are boycotting over increasing medical school places. Perspect. Med. Educ..

[23] Cho S.I., Lee J.M., Park H.J., Suh J., Lee R.W. (2025). Healthcare crisis in Korea and its impact on medical research: A PubMed analysis (2022–2024). J. Korean Med. Sci..

[24] Lee K., Seong B.O., Yoo M.W. (2024). Comparison of the postoperative complications for gastric cancer surgery before and during the medical crisis in South Korea: A retrospective observational study. Ann. Surg. Treat. Res..

[25] Park D., Jeong E., Lee S.Y., Kim M., Hong D.Y., Kwon H.D., Kim M.C. (2022). Behavioral and disease-related characteristics of patients with acute stroke during the coronavirus disease pandemic. Healthcare.

[26] Kang J., Song H., Kim S.E., Kim J.Y., Park H.K., Cho Y.J., Lee K.B., Lee J., Lee J.S., Choi A.R. (2024). Network analysis of stroke systems of care in Korea. BMJ Neurol. Open.

[27] Jeong J.-O. (2013). Regional cardiocerebrovascular center project in the treatment of acute myocardial infarction. Korean J. Med..

[28] Miller A.O., Kapadia M., Kirksey M.A., Sandhu M., Jannat-Khah D., Bui T., Boyle K.K., Krez A., Russell L., O’Neill J. (2020). Clinical experience with COVID-19 at a specialty orthopedic hospital converted to a pandemic overflow field hospital. HSS J..

[29] Haileamlak A. (2021). The impact of COVID-19 on health and health systems. Ethiop. J. Health Sci..

[30] Rudilosso S., Laredo C., Vera V., Vargas M., Renu A., Llull L., Obach V., Amaro S., Urra X., Torres F. (2020). Acute stroke care is at risk in the era of COVID-19: Experience at a comprehensive stroke center in Barcelona. Stroke.

[31] Hoyer C., Ebert A., Huttner H.B., Puetz V., Kallmunzer B., Barlinn K., Haverkamp C., Harloff A., Brich J., Platten M. (2020). Acute stroke in times of the COVID-19 pandemic: A multicenter study. Stroke.

[32] Kim S.J., Kim S.J., Han K.T., Park E.C. (2017). Medical costs, Cesarean delivery rates, and length of stay in specialty hospitals vs. non-specialty hospitals in South Korea. PLoS ONE.

[33] Swiezewski S.P., Rzonca P., Panczyk M., Leszczynski P.K., Gujski M., Michalak G., Fronczak A., Galazkowski R. (2019). Polish helicopter emergency medical service (HEMS) response to stroke: A five-year retrospective study. Med. Sci. Monit..

[34] Xu H., Xian Y., Woon F.P., Bettger J.P., Laskowitz D.T., Ng Y.Y., Ong M.E.H., Matchar D.B., De Silva D.A. (2020). Emergency medical services use and its association with acute ischaemic stroke evaluation and treatment in Singapore. Stroke Vasc. Neurol..

[35] Menendez Albarracin A., Valls Carbo A., Rabaneda Lombarte N., Yugueros Baena B., Carbonell Gisbert J., Flores-Pina B., Larranaga De Bofarull M.C., Martinez Sanchez M., Hernandez-Perez M., Bustamante Rangel A. (2024). Time of the day and season distribution among stroke code subtypes: Differences between ischemic stroke, intracranial hemorrhage, and stroke mimic. Front. Neurol..

