# Supplementary material for: Impact of the 2024 Resident Physician Work Stoppage on Acute Hemorrhagic Stroke Admissions: A Single Cerebrovascular-Specialty Hospital Study in South Korea
Source: Healthcare (Basel). 2025 Aug 27;13(17):2129. doi: 10.3390/healthcare13172129 (PMC12428150; doi:10.3390/healthcare13172129)
Supplement: Supplementary file 1 [file healthcare-13-02129-s001.zip › healthcare-3740088-supplementary.pdf]

Table S1. Initial vital signs and laboratory findings of the patients.

Variables	BC group (n = 130)	AC group (n = 214)	*p*-value
Systolic blood pressure, mmHg	159.0 (145.0–186.0)	162.0 (146.0–183.0)	0.903
Diastolic blood pressure, mmHg	95.0 (81.0–107.0)	94.0 (83.0–107.0)	0.673
Heart rate, n/minute	75.5 (68.0–90.0)	77.0 (66.0–88.0)	0.945
Body temperature, ℃	36.5 (36.5–36.6)	36.5 (36.5–36.6)	0.150
White blood cell, 10^3/μL	8.9 (6.7–12.5)	9.0 (6.8–12.0)	0.736
Hemoglobin, g/dL	13.3 (11.9–14.7)	13.6 (12.4–14.7)	0.225
Platelet, 10^3/μL	226.0 (182.0–270.0)	229.0 (193.0–278.0)	0.248
Random glucose, mg/dL	138.0 (116.0–170.0)	142.0 (117.0–180.0)	0.704
C-reactive protein, mg/L	0.1 (0.1–0.3)	0.1 (0.0–0.3)	0.755

Abbreviations: AC, after crisis; BC, before crisis.

Table S2. Regional distribution and time-related parameters after propensity score matching.

Variables	BC group (n = 130)	AC group (n =130)	*p*-value
Regional categories, n (%)			<0.001
Within Pohang city	63 (48.5)	42 (32.3)	
Within the region	54 (41.5)	40 (30.8)	
Within Daegu/Gyeongsangbuk province	8 (6.2)	43 (33.1)	
Outside Daegu/Gyeongsangbuk province	5 (3.8)	5 (3.8)	
Onset to arrival, minutes	95.0 (57.0–281.0)	134.0 (63.0–257.0)	0.221
Onset to operation, minutes	166.0 (125.0–235.0)	200.0 (137.5–285.0)	0.130
Arrival at initial imaging, minutes	16.0 (11.0–28.0)	15.0 (11.0–24.0)	0.399
Arrival to operation, minutes	65.0 (54.0–79.0)	66.0 (58.0–82.5)	0.529

Abbreviations: AC, after crisis; BC, before crisis.

Table S3. Characteristics of patients with subarachnoid hemorrhage.

Variables	BC group (n = 29)	AC group (n = 70)	*p*-value
Age, years	61.6 ± 11.3	61.1 ± 13.6	0.875
Male, n (%)	8 (27.6)	20 (28.6)	>0.999
Body mass index, kg/m^2	23.7 (20.9–26.0)	23.9 (21.3–26.2)	0.432
Regional categories, n (%)			0.018
Within Pohang city	12 (41.4)	24 (34.3)	
Within the region[a]	15 (51.7)	20 (28.6)	
Within Daegu/Gyeongsangbuk province[b]	2 (6.9)	23 (32.9)	
Outside Daegu/Gyeongsangbuk province	0 (0.0)	3 (4.3)	
Visit types, n (%)			0.807
First visit	16 (55.2)	34 (48.6)	
Transferred from another hospital[c]	1 (3.4)	2 (2.9)	
Referred from another hospital[d]	12 (41.4)	34 (48.6)	
Initial GCS score	15.0 (8.0–15.0)	15.0 (13.0–15.0)	0.370
NIHSS	0.0 (0.0–19.0)	0.0 (0.0–6.0)	0.451
Operation, n (%)	28 (96.6)	61 (87.1)	0.295
Onset to arrival, minutes	115.0 (68.0–313.0)	141.5 (69.0–336.0)	0.617
Onset to operation, minutes	211.0 (132.5–427.5)	235.0 (135.0–325.0)	0.891
Arrival at initial imaging, minutes	13.0 (11.0–21.0)	14.0 (10.0–21.0)	0.726
Arrival to operation, minutes	68.0 (54.5–86.0)	60.0 (46.0–73.0)	0.069
Hospital stay, days	24.0 (17.0–35.0)	23.0 (16.0–33.0)	0.610
Current smoker, n (%)	7 (24.1)	19 (27.1)	0.954
Comorbidities, n (%)			
Hypertension	12 (41.4)	26 (37.1)	0.867
Diabetes	4 (13.8)	8 (11.4)	>0.999
Dyslipidemia	6 (20.7)	14 (20.0)	>0.999
Coronary artery diseases	2 (6.9)	2 (2.9)	0.713
Cerebrovascular accidents	6 (20.7)	4 (5.7)	0.060
Modified Rankin scale at 3 months, n (%)			0.215
0	10 (37.0)	17 (24.6)	
1	7 (25.9)	32 (46.4)	
2	2 (7.4)	4 (5.8)	
3	3 (11.1)	1 (1.4)	
4	2 (7.4)	8 (11.6)	
5	1 (3.7)	1 (1.4)	
6	2 (7.4)	6 (8.7)	

[a]Gyeongju, Yeongdeok, and Uljin.

[b]excluding the Gyeongju, Yeongdeok, and Uljin areas.

[c]≥6 hours stay

[d]<6 hours stay

Abbreviations: AC, after crisis; BC, before crisis; GCS, Glasgow Coma Scale; NIHSS, National Institutes of Health Stroke Scale.

Table S4. Characteristics of patients without any operative care.

Variables	BC group (n = 53)	AC group (n = 88)	*p*-value
Age, years	66.2 ± 15.5	64.5 ± 13.4	0.497
Male, n (%)	29 (54.7)	49 (55.7)	>0.999
Body mass index, kg/m^2	22.5 (19.6–24.8)	24.0 (21.8–26.0)	0.028
Primary diagnosis, n (%)			0.126
Intracranial hemorrhage	52 (98.1)	79 (89.8)	
Subarachnoid hemorrhage	1 (1.9)	9 (10.2)	
Regional categories, n (%)			0.028
Within Pohang city	27 (50.9)	34 (38.6)	
Within the region[a]	22 (41.5)	29 (33.0)	
Within Daegu/Gyeongsangbuk province[b]	3 (5.7)	22 (25.0)	
Outside Daegu/Gyeongsangbuk province	1 (1.9)	3 (3.4)	
Visit types, n (%)			0.016
First visit	34 (64.2)	63 (71.6)	
Transferred from another hospital[c]	8 (15.1)	2 (2.3)	
Referred from another hospital[d]	11 (20.8)	23 (26.1)	
Initial GCS score	15.0 (14.0–15.0)	15.0 (14.0–15.0)	0.970
NIHSS	3.0 (1.0–9.0)	3.5 (1.0–11.0)	0.950
Onset to arrival, minutes	162.0 (72.0–301.0)	142.5 (76.5–337.5)	0.792
Arrival at initial imaging, minutes	21.0 (14.0–37.0)	20.0 (12.0–37.0)	0.650
Hospital stay, days	17.5 (10.5–29.5)	14.0 (9.0–22.0)	0.079
Current smoker, n (%)	15 (28.3)	22 (25.3)	0.846
Comorbidities, n (%)			
Hypertension	25 (47.2)	43 (48.9)	0.983
Diabetes	9 (17.0)	21 (23.9)	0.450
Dyslipidemia	6 (11.3)	20 (22.7)	0.142
Coronary artery diseases	4 (7.5)	5 (5.7)	0.934
Cerebrovascular accidents	6 (11.3)	14 (15.9)	0.612
Modified Rankin scale at 3 months, n (%)			0.176
0	5 (10.0)	6 (8.0)	
1	12 (24.0)	35 (46.7)	
2	10 (20.0)	8 (10.7)	
3	7 (14.0)	5 (6.7)	
4	8 (16.0)	10 (13.3)	
5	4 (8.0)	3 (4.0)	
6	4 (8.0)	8 (10.7)	

[a]Gyeongju, Yeongdeok, and Uljin.

[b]excluding the Gyeongju, Yeongdeok, and Uljin areas.

[c]≥6 hours stay

[d]<6 hours stay

Abbreviations: AC, after crisis; BC, before crisis; GCS, Glasgow Coma Scale; NIHSS, National Institutes of Health Stroke Scale.
